# Prevalence of Digital Eye Strain Symptoms Among Sudanese Medical Students During Conflict‐Induced Online Learning: A Cross‐Sectional Study

**DOI:** 10.1002/hsr2.73029

**Published:** 2026-08-09

**Authors:** Moram Elfadel Abdelrhaman Gasmalha, Israa Alamin Mohammed Hussein, Olla Zuhair Elamin Abdalla, Blsam Abdelmoneam Ahmed Mohammed, Safinat Hassan Mohammed Ahmed, Azza Zuhair Elamin Abdalla, Sajda Ibrahim Abood Almatri, Asia Moawia Mirgani Hassan, Abdelmoula Hashim Abdelmagid Mohamed, Yousif Abdelrahman Yousif Fadlelmoula, Mohamed Salah Abdulrazeg Abdulrahman, Sohaib Mohammed Mokhtar Ahmed

**Affiliations:** ^1^ Faculty of Medicine and Health Sciences University of Omdurman Islamic Omdurman Sudan; ^2^ Faculty of Pharmacy University of Gadarif Gadarif Sudan; ^3^ Faculty of Medicine and Surgery Sudan International University Khartoum Sudan; ^4^ Faculty of Medicine and Health Sciences Red Sea University Port Sudan Sudan; ^5^ Faculty of Medicine University of Gezira Wad Medani Sudan; ^6^ Faculty of Dentistry University of Gezira Wad Medani Sudan; ^7^ Faculty of Medicine Alzaiem Alazhari University Khartoum Sudan; ^8^ Faculty of Medicine and Health Sciences University of Gadarif Gadarif Sudan

**Keywords:** computer vision syndrome, digital eye strain, medical students, online learning, Sudan

## Abstract

**Background and Aims:**

Armed conflict in Sudan has severely disrupted traditional medical education, prompting a shift toward online learning as an alternative. While digital platforms enable educational continuity, they are associated with increased screen exposure and a rising incidence of digital eye strain (DES) among medical students. This study aimed to investigate the prevalence, symptoms, and associated factors of DES in this conflict‐driven educational environment. To assess the prevalence, risk factors, and awareness of DES symptoms among Sudanese medical students engaged in online learning during the armed conflict.

**Methods:**

A descriptive cross‐sectional study was conducted from April to June 2025 among undergraduate Sudanese medical students who transitioned to online learning during the conflict. A total of 1028 participants were recruited using convenience sampling. Data were collected using an online self‐administered questionnaire adapted from a validated instrument used in a previously published study. Responses were analyzed using SPSS version 27. Descriptive statistics were used to summarize findings, binomial logistic regression was performed to identify predictors of DES symptoms, and the Wilcoxon signed‐rank test assessed changes in screen time before and during the conflict.

**Results:**

About 80.6% of students reported that prolonged screen use negatively impacted their eye health and lifestyle. Eye‐related symptoms were reported by 86.6% of participants, and 61.5% experienced physical discomfort. The average screen time increased significantly during the conflict. Awareness of the 20‐20‐20 rule was limited (24.2%), with only 13.5% reporting regular practice. However, 70.9% were willing to reduce screen time to help prevent DES.

**Conclusion:**

Digital eye strain was highly prevalent among medical students and was associated with extended screen use, poor posture, and limited awareness. Its association with physical discomfort and academic stress highlights the need for targeted interventions. Medical institutions should integrate DES awareness and prevention strategies into curricula to promote student well‐being and academic success.

**Abbreviations**
AOA: American optometric association
CI: confidence interval
CVS: computer vision syndrome
DES: digital eye strain
OR: odds ratio
SD: standard deviation
SPSS: statistical package for the social sciences

## 1 Introduction

The recent rise in technology use has made online learning a common form of education, especially in areas affected by pandemics and armed conflicts [1, 2]. In Sudan, the conflict that erupted on April 15, 2023, led to the suspension of traditional medical education due to several factors, including the destruction of infrastructure, forced displacement, and ongoing security threats. In such circumstances, online learning emerged as a viable alternative, allowing universities to maintain educational continuity while ensuring the safety of students [3].

Online learning, facilitated by computers, smartphones, and various digital devices, has significantly transformed modern education by providing flexibility and accessibility across multiple platforms [4, 5]. However, prolonged screen exposure during online learning can negatively affect students' health. One of the recognized consequences is digital eye strain (DES), also known as computer vision syndrome (CVS).

According to the American Optometric Association (AOA), DES refers to a group of vision‐related and ocular symptoms resulting from prolonged use of digital devices [6]. DES symptoms are commonly classified into three main categories. Visual symptoms include blurred vision, visual fatigue, and diplopia. Ocular symptoms include dryness, redness, irritation, and eye strain. Extraocular symptoms involve headaches, as well as neck, shoulder, or back pain [7]. Various factors contribute to the development of DES, including poor lighting conditions, excessive screen glare, inappropriate viewing distances, inadequate ergonomic posture, and uncorrected refractive errors [6].

Globally, approximately 60 million individuals are affected by DES, with an additional 1 million new cases occurring annually. The COVID‐19 pandemic resulted in a substantial increase in screen exposure due to lockdowns and remote learning, with internet usage increasing by 40% to 100% [8]. During this period, several studies reported a high prevalence of DES across different populations, particularly among students engaged in online learning. A study among undergraduate medical students in Kerala, India, found that 90.3% of participants experienced ocular and extraocular symptoms, mainly neck pain, headaches, and watery eyes [9]. Similarly, a study conducted among undergraduate medical students in Haryana, India, reported that 88.02% of participants experienced at least one DES symptom, while 76.77% reported at least one non‐ocular symptom [10]. Consistent with these findings, a population‐based study in India involving students, teachers, and members of the general public found that eye strain was significantly more prevalent among students attending online classes compared with the general population (50.6% vs. 33.2%) [11]. Likewise, a study among medical students at King Abdulaziz University in Jeddah, Saudi Arabia, reported that 95% of participants experienced at least one symptom of computer vision syndrome (CVS) during computer use for studying [12]. Additionally, a study conducted among medical students in Nepal reported an overall DES prevalence of 90.8% among participants who experienced at least one ocular or non‐ocular symptom [13]. Furthermore, a study conducted among clinical‐year medical students at the University of Bahri, Khartoum State, Sudan, reported a high prevalence of DES and an association between greater symptom severity and lower academic performance [14].

To our knowledge, this is the first study assessing DES among medical students in a conflict‐affected educational setting. Unlike previous studies conducted in stable environments, this study captures the combined effects of war, displacement, and increased digital device use. Therefore, this study aimed to assess the prevalence, associated factors, and awareness of DES symptoms among Sudanese medical students engaged in online learning during the armed conflict.

## 2 Materials and Methods

### 2.1 Study Design and Setting

A cross‐sectional study was conducted in April and May 2025 among undergraduate medical students from both public and private universities in Sudan. The study population comprised students who transitioned to online learning as a result of the ongoing conflict in Sudan. The primary objective was to assess the prevalence, risk factors, symptoms, and awareness of DES among this cohort.

### 2.2 Study Population

Participants were Sudanese medical students enrolled in institutions that adopted online education during the conflict. They were specifically selected because the transition to online learning during the conflict substantially increased their reliance on digital devices for academic activities, placing them at increased risk of digital eye strain. The inclusion criteria were: (1) current enrollment in a medical program; (2) exposure to online learning during the conflict period; and (3) consent to participate. Students who continued exclusively with traditional (in‐person) education during the conflict were excluded. Additionally, students with pre‐existing eye conditions were excluded from the study.

### 2.3 Sample Size and Sampling Technique

Due to the absence of reliable national data on the total number of medical students in Sudan, the sample size was calculated using Cochran's formula for an unknown population. A 98% confidence level was selected to increase the precision of the sample size estimation and reduce the margin of error. Using a 4% margin of error and an assumed response distribution of 50%, the minimum required sample size was calculated to be 849 students. To account for potential limitations related to representativeness and anticipated non‐response, the target sample size was increased, resulting in a final sample of 1028 students.

Convenience sampling was employed through the dissemination of the survey via online platforms (WhatsApp, Facebook, and Telegram) because of the logistical constraints and limited physical access caused by the conflict.

### 2.4 Data Collection Procedure and Instrument

Data were collected using a structured, self‐administered online questionnaire developed via Google Forms. The questionnaire was adapted from a validated tool used in a prior study in India [15]. It comprised four sections:

Section 1: Socio‐demographic characteristics, including age, gender, place of residence, academic year, living arrangement, marital status, and socioeconomic status.

Section 2: Screen use patterns, covering the type and number of devices used, average screen time before and during the conflict, nature of usage (continuous when participants used digital devices for prolonged periods without regular breaks and intermittent when screen use was interrupted by periodic breaks), time of use (day vs. night), and post‐screen visual perception (e.g., blurred, clear, or hazy vision).

Section 3: DES symptoms, assessing self‐reported symptoms such as eye strain, headache, dry eyes, double vision, and neck or shoulder pain, along with associated refractive errors, use of corrective glasses, and anti‐reflective coatings.

Section 4: Awareness of DES, evaluating participants' knowledge of the condition, understanding of the 20‐20‐20 rule, and willingness to modify screen use behavior.

The tool was piloted on 20 medical students for clarity and cultural relevance. Minor adjustments were made to phrasing. The finalized survey was distributed through widely used academic and extracurricular medical student networks on social media.

### 2.5 Data Management and Analysis

All data were analyzed using SPSS version 27. Descriptive statistics (frequencies and percentages) were used to summarize demographic variables, screen usage patterns, symptom prevalence, refractive error status, and DES awareness. The Wilcoxon signed‐rank test was applied to compare self‐reported screen time before and during the conflict period. Binomial logistic regression analysis was conducted to identify significant predictors of DES‐related symptoms.

### 2.6 Variables

The primary outcome variable was the presence of DES symptoms. Independent variables included participants' sociodemographic characteristics, digital device use patterns, awareness and practice of the 20‐20‐20 rule, perceived impact of screen use, and self‐reported ocular and physical symptoms. These variables were analyzed to identify factors associated with DES symptoms.

### 2.7 Ethics Approval and Consent to Participate

Ethical approval was obtained from the Medical Research Ethics Committee, Faculty of Medicine and Health Sciences, University of Gadarif, under reference number (Ref. No.:GU/FM/REC/Q2.05.25.5). Written Informed consent was obtained from all participants prior to their involvement in the study. Participants were informed of their right to withdraw at any stage without consequences. Confidentiality and anonymity were strictly maintained throughout the research process.

## 3 Results

### 3.1 Participant Characteristics

A total of 1028 medical students participated in the study. The mean age was 22.1 years (SD ± 2.5; range 18–35). Females comprised 68.5% (*n* = 704) of the sample. Regarding the current place of residence during the study period, 43.8% lived in urban areas, 23.6% in rural areas, and 32.6% were residing outside Sudan. Distribution across academic years was: first year 8.6% (*n* = 88), second 31.1% (*n* = 320), third 20.0% (*n* = 206), fourth 20.0% (*n* = 206), fifth 13.9% (*n* = 143), and sixth 6.3% (*n* = 65). Most participants (79.4%, *n* = 816) lived with family; others lived alone (5.1%, *n* = 52), with relatives (4.9%, *n* = 50), or with roommates (10.7%, *n* = 110). Nearly all were single (96.5%, *n* = 992). Participants' self‐perceived household economic status was predominantly middle (64.7%, *n *= 665), with small proportions rating themselves as high (2.2%, *n* = 23) or low (3.8%, *n *= 39). Regarding devices for online learning, 46.4% (*n *= 477) used one device, 44.4% (*n * = 456) used two, and 9.2% (*n *= 95) used three or more. Smartphones were the most frequently used digital screen (76.4%, *n *= 785), followed by tablets/iPads (10.7%, *n *= 110) and laptops (12.9%, *n *= 133) (Table 1).

**Table 1:** Socio‐demographic characteristics of the participants (*N* = 1028).

Variables	Overall (*N* = 1028)
Age:	
Mean (SD)	22.1 (2.5)
Range	18.0–35.0
Sex:	
Female	704 (68.5%)
Male	324 (31.5%)
Residence:	
Urban area	450 (43.8%)
Rural area	243 (23.6%)
Outside Sudan	335 (32.6%)
Year of study:	
First year	88 (8.6%)
Second year	320 (31.1%)
Third year	206 (20.0%)
Fourth year	206 (20.0%)
Fifth year	143 (13.9%)
Sixth year	65 (6.3%)
Living situation:	
Living alone	52 (5.1%)
Living with family	816 (79.4%)
Living with relatives	50 (4.9%)
Living with roommates	110 (10.7%)
Marital status:	
Single	992 (96.5%)
Married	34 (3.3%)
Divorced	1 (0.1%)
Widowed	1 (0.1%)
Self‐rated economic status:	
High	23 (2.2%)
Upper‐middle	157 (15.3%)
Middle	665 (64.7%)
Lower‐middle	144 (14.0%)
Low	39 (3.8%)
Number of devices for online learning:	
One	477 (46.4%)
Two	456 (44.4%)
Three or more	95 (9.2%)
The frequent computer digital screen:	
Smart phone	785 (76.4%)
Tablet or iPad	110 (10.7%)
Laptop	133 (12.9%)

### 3.2 Digital Screen Usage Patterns and Behavioral Characteristics

Continuous screen use (without regular breaks) was reported by 37.3% (n = 383) of students, while 62.7% (*n* = 645) used screens intermittently. Participants reported their predominant period of screen use, with 60.3% indicating mainly daytime use and 39.7% mainly nighttime use. After prolonged screen exposure, 56.9% (*n* = 585) maintained clear vision, whereas 33.5% (*n* = 344) experienced blurred vision and 9.6% (*n* = 99) reported haziness. Self‐reported refractive error was acknowledged by 16.8% (*n* = 173), with 41.8% (*n* = 430) denying any error and 41.3% (*n* = 425) uncertain. Vision correction usage comprised glasses (36.5%, *n* = 375) and contact lenses (3.4%, *n* = 35); 60.1% (*n* = 618) used no correction. Anti‐reflective coating glasses were used by 24.9% (*n* = 256). Only 24.2% (*n* = 249) were aware of the 20‑20‑20 rule, and a mere 13.5% (*n* = 139) practiced it. However, 70.9% (*n* = 729) expressed willingness to reduce screen time to prevent DES (Table 2).

**Table 2:** Digital screen usage patterns and behavioral characteristics (*n* = 1028).

Item	Category	*n* (%)
Screen time pattern	Continuous	383 (37.3%)
	Interrupted	645 (62.7%)
Predominant time of screen use	Day	620 (60.3%)
	Night	408 (39.7%)
Visual clarity after prolonged screen time	Clear	585 (56.9%)
	Blurred	344 (33.5%)
	Hazy	99 (9.6%)
Self‐reported refractive error	Yes	173 (16.8%)
	No	430 (41.8%)
	I don't know	425 (41.3%)
Vision correction usage	Contact lens	35 (3.4%)
	Glasses	375 (36.5%)
	None	618 (60.1%)
Use of anti‐refractive coating glasses	Yes	256 (24.9%)
	No	772 (75.1%)
Awareness of 20‐20‐20 rule	Yes	249 (24.2%)
	No	779 (75.8%)
Practice of 20‐20‐20 rule	Yes	139 (13.5%)
	No	889 (86.5%)
Willingness to reduce screen time to prevent CVS	Yes	729 (70.9%)
	No	299 (29.1%)

### 3.3 Self‐Reported Health Effects After Prolonged Screen Exposure

**Figure 1:**
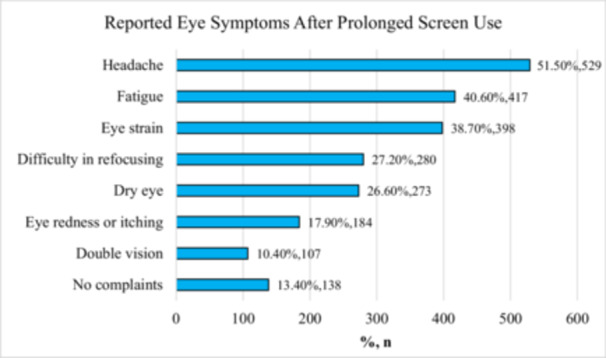
Prevalence of self‐reported eye symptoms after prolonged screen use.

**Figure 2:**
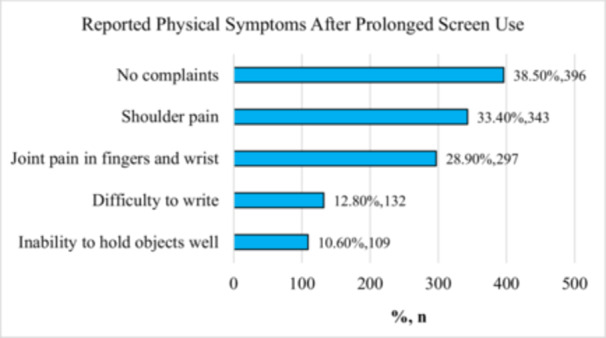
Prevalence of self‐reported physical symptoms after prolonged screen use.

A majority of participants (80.6%, *n *= 829) perceived that digital screen use adversely affected their eye health and lifestyle. Self‐reported eye symptoms—such as dryness, itching, or redness—were noted by 86.6% (*n *= 890), and physical symptoms—such as joints pain or musculoskeletal discomfort—by 61.5% (*n *= 632) (Table 3; Figures 1 and 2).

**Table 3:** Self‐reported health effects after prolonged screen exposure (*n* = 1028).

Item	Yes (*n*, %)	No (*n*, %)
Perception that screen use affects eye health and lifestyle	829 (80.6%)	199 (19.4%)
Self‐reported eye symptoms after prolonged digital screen use	890 (86.6%)	138 (13.4%)
Self‐reported physical symptoms after prolonged digital screen use	632 (61.5%)	396 (38.5%)

### 3.4 Change in Screen Time Pre‐ vs. During Conflict

**Figure 3:**
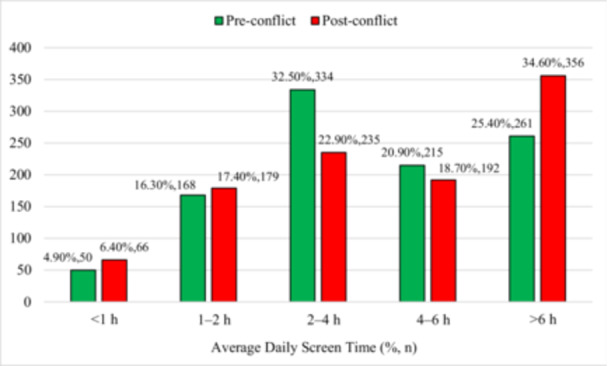
Comparison of average daily screen time before and during the conflict (in hours).

Average daily digital screen time increased significantly during the conflict. Before the conflict, mean screen time was 4.32 h (SD ± 2.47; median = 3 h), rising to 4.68 h (SD ± 2.71; median = 5 h) during the conflict. A Wilcoxon signed‐rank test confirmed this increase was statistically significant (*W *= 64,791; *p *< 0.001). The average paired difference was −0.75 h (95% CI: –1.00 to –0.25), with a small‐to‐moderate effect size (rank biserial correlation = –0.239) (Figure 3).

### 3.5 Predictors of Eye Symptoms

Binomial logistic regression identified several significant predictors of self‐reported eye symptoms (Table 4). Compared to first‐year students, those in third (OR = 2.86; 95% CI: 1.25–6.55; *p *= 0.013), fourth (OR = 2.52; 95% CI: 1.09–5.82; *p *= 0.031), and fifth years (OR = 2.77; 95% CI: 1.08–7.08; *p *= 0.034) had higher odds of reporting eye symptoms. Blurred vision after prolonged screen use was associated with increased odds (OR = 2.47; 95% CI: 1.47–4.16; *p *< 0.001), as was hazy vision (OR = 2.46; 95% CI: 1.06–5.72; *p *= 0.037). Participants reporting physical symptoms had over four times the odds of eye symptoms (OR = 4.25; 95% CI: 2.76–6.56; *p *< 0.001). Interestingly, students who perceived that screen use did not affect their eye health or lifestyle were less likely to report symptoms (OR = 0.34; 95% CI: 0.22–0.54; *p *< 0.001). No other predictors reached statistical significance at *p *< 0.05.

**Table 4:** Binomial logistic regression analysis predicting presence of eye symptoms among medical students (*n* = 1028).

Predictor	*p*	Odds ratio (OR)	95% CI lower	95% CI upper
Intercept	0.071	8.57	0.83	88.41
Age	0.259	0.94	0.85	1.04
Year of study				
Second year vs. first year	0.648	1.18	0.58	2.42
Third year vs. first year	0.013*	2.86	1.25	6.55
Fourth year vs. first year	0.031*	2.52	1.09	5.82
Fifth year vs. first year	0.034*	2.77	1.08	7.08
Sixth year vs. first year	0.266	1.84	0.63	5.41
Digital time usage in the pre‐conflict	0.059	0.92	0.83	1.00
Digital time usage during conflict period	0.057	1.09	1.00	1.19
Hours spent on digital screen (interrupted vs. continuous)	0.315	0.80	0.51	1.24
Screen time mostly (night vs. day)	0.473	0.86	0.57	1.30
Detail of objects seen (blurred vs. clear)	< 0.001*	2.47	1.47	4.16
Detail of objects seen (hazy vs. clear)	0.037*	2.46	1.06	5.72
Physical symptoms (yes vs. no)	< 0.001*	4.25	2.76	6.56
Use of antirefractive coating glasses (no vs. yes)	0.092	0.63	0.36	1.08
Willingness to decrease screen hours (no vs. yes)	0.568	1.14	0.73	1.77
Refractive error (no vs. yes)	0.746	0.89	0.45	1.77
Refractive error (don't know vs. yes)	0.436	1.33	0.65	2.70
Digital screen affects lifestyle/eye health (no vs. yes)	< 0.001*	0.34	0.22	0.54
Awareness of the 20‐20‐20 rule (no vs. yes)	0.192	1.37	0.85	2.19

*Note:* Model fit statistics: Nagelkerke *R* ^2 ^= 0.287; Hosmer–Lemeshow *χ* ^2 ^= 8.14, *p* = 0.420; overall classification accuracy = 73.6% (sensitivity 68.2%, specificity 77.1%); AIC = 462.3.

* indicates statistical significance at *p *< 0.05.

## 4 Discussion

This study revealed key findings concerning DES among medical students during conflict. First, screen time significantly increased during the conflict period. Second, the prevalence of self‐reported DES symptoms was extremely high (86.6%), with over 60% also experiencing physical symptoms. Third, knowledge and application of the 20‐20‐20 preventive rule were remarkably low. Fourth, there was a strong positive association between physical and visual complaints. Finally, students in advanced academic years had significantly higher odds of experiencing DES symptoms. These findings highlight a substantial public health issue with practical, clinical, and educational consequences that demand urgent attention.

The significant increase in daily screen time during conflict (from 4.32 to 4.68 h, *p *< 0.001) is likely driven by a combination of enforced digital learning modalities, limited outdoor access, and reduced non‐screen‐based activities. Similar behavioral patterns have been documented during other crises, such as the COVID‐19 pandemic, where lockdowns drastically increased reliance on digital platforms for education, communication, and entertainment [16, 17]. Real‐time sensing studies from the 2021 Israel–Gaza conflict similarly found increased screen exposure during periods of crisis [18]. Elevated screen time is directly linked to higher risks of ocular and systemic strain [17]. Therefore, periods of sociopolitical instability should be recognized as high‐risk environments for digital overuse, necessitating proactive digital health strategies and institutional planning.

The prevalence of DES symptoms in this cohort (86.6%) is consistent with reports from comparable studies during the pandemic, which found rates of 68.53% to 90.3% in university and medical students [19, 20]. The consistency across contexts highlights that young adults, especially those in high cognitive load environments, are particularly vulnerable to screen‐induced symptoms such as eye fatigue, burning, and blurred vision. These symptoms are the core components of Computer Vision Syndrome, which results from prolonged near work and decreased blink rate [21]. If left unaddressed, chronic DES may lead to long‐term accommodative dysfunction and reduced academic performance. Institutions must therefore consider periodic screening, policy‐regulated screen breaks, and incorporation of ocular hygiene practices into student health programs.

The extremely low awareness (24.2%) and practice (13.5%) of the 20‐20‐20 rule reflects a major educational gap, despite its wide promotion in clinical and public health guidelines. Similar levels of poor adherence have been noted in studies from India and Nepal, where even among medical students and professionals, awareness remained below 40%, and actual compliance even lower [13, 15]. This disconnect between awareness campaigns and actual behavior may stem from a lack of institutional enforcement or inadequate emphasis on eye health in student wellness curricula. Without behavioral change, the risk of cumulative damage from unbroken screen use persists. Short digital wellness modules or enforced microbreaks in virtual classrooms may bridge this gap and encourage protective habits.

The strong association between physical complaints (e.g., neck/back pain, headaches) and DES symptoms (OR = 4.25) indicates a broader syndrome of digital ergonomics failure, not just isolated ocular fatigue. Similar associations have been documented in both student and office worker populations, where musculoskeletal strain commonly co‐occurs with visual complaints due to poor posture, suboptimal workstation setup, and uninterrupted near‐focus tasks [22, 23]. These findings argue for a combined ergonomic intervention strategy rather than a vision‐only focus. Failure to address co‐occurring symptoms could lead to increased absenteeism, reduced academic productivity, and chronic pain syndromes. Introducing ergonomic assessments and posture training in academic settings is a logical, low‐cost preventive measure.

Finally, the finding that third‐ to fifth‐year students had 2.5–2.9 times greater odds of DES compared to first‐year students aligns with research indicating cumulative screen exposure and academic intensity as major risk factors [24]. Older students typically face heavier workloads, more screen‐based study, and longer hours of continuous focus, all contributing to increased strain [24]. The progressive nature of DES risk reinforces the need for early education and preventive action. Waiting until symptoms are severe in the senior years is inefficient and costly. Tailored interventions should begin early in the medical curriculum and scale in complexity and intensity as students progress through their training.

### 4.1 Implications and Recommendations

The high prevalence of digital eye strain symptoms among medical students, along with increased screen time during conflict and poor adoption of preventive practices, indicates a pressing need for practical, scalable interventions. These should begin with integrating basic eye health education and ergonomic principles into the early years of medical training, especially as senior students are more affected. Promoting low‐cost, evidence‐based strategies such as the 20‐20‐20 rule, scheduled screen breaks, and awareness campaigns within the academic setting is both feasible and necessary. Given the strong association between physical and ocular symptoms, interventions should also address workstation ergonomics and posture, not just visual habits. Institutional policies must be adapted to recognize increased digital exposure during crises, and student health services should consider routine screening for DES symptoms. These actions can be realistically implemented within existing academic structures to reduce symptom burden, preserve student productivity, and prevent long‐term consequences.

## 5 Limitations

This study has several limitations. First, its cross‐sectional design limits the ability to establish casual relationship; while associations between screen time, symptoms, and academic factors were identified, temporal relationships cannot be confirmed, and the observed associations should not be interpreted as evidence of causality. Second, all data were self‐reported, introducing the potential for recall bias and subjective misclassification of symptoms, screen time, and preventive behaviors. Third, no objective ophthalmologic or ergonomic assessments were performed to validate symptom reports or screen exposure. Fourth, the sample was restricted to medical students, which may limit generalizability to other student populations or working professionals. Fourth, the study employed a convenience sampling approach and was restricted to medical students, which may have introduced selection bias and limited the generalizability of the findings. In addition, the response rate could not be calculated because of the sampling approach.

Lastly, the study was conducted during an ongoing conflict, which may have uniquely influenced behavior, lifestyle, and health perceptions in ways that differ from stable contexts. Future longitudinal and interventional studies are needed to better understand causality, validate findings, and evaluate the effectiveness of targeted preventive strategies.

## 6 Conclusion

This study demonstrates that digital eye strain is highly prevalent among medical students, worsened by crisis‐induced increases in screen time and compounded by poor ergonomic habits and low awareness of preventive strategies. The significant association between DES, physical discomfort, and academic seniority illustrates the multifaceted nature of the issue. Without urgent educational, clinical, and policy interventions, the burden of DES will continue to grow—undermining both student well‐being and academic performance. Comprehensive, level‐specific strategies are needed to integrate ocular health into the broader framework of medical education and public health preparedness.

## Author Contributions

**Moram Elfadel Abdelrhaman Gasmalha:** conceptualization, methodology, data curation, writing – original draft, project administration, resources, supervision, investigation, writing – review and editing. **Israa Alamin Mohammed Hussein:** writing – original draft, software, formal analysis, investigation. **Olla Zuhair Elamin Abdalla:** writing – original draft, resources, software. **Blsam Abdelmoneam Ahmed Mohammed:** writing – original draft, data curation, project administration. **Safinat Hassan Mohammed Ahmed:** writing – original draft, data curation, resources. **Azza Zuhair Elamin Abdalla:** writing – original draft, resources, investigation. **Sajda Ibrahim Abood Almatri:** writing – original draft, data curation. **Asia Moawia Mirgani Hassan:** writing – original draft, data curation. **Abdelmoula Hashim Abdelmagid Mohamed:** writing – original draft, data curation. **Yousif Abdelrahman Yousif Fadlelmoula:** software, formal analysis, writing – original draft, investigation. **Mohamed Salah Abdulrazeg Abdulrahman:** writing – review and editing, investigation, writing – original draft. **Sohaib Mohammed Mokhtar Ahmed:** supervision, visualization, writing – review and editing, formal analysis, writing – original draft.

## Funding

The authors have nothing to report.

## Conflicts of Interest

The authors declare no conflicts of interest.

## Transparency Statement

Moram Elfadel, the manuscript guarantor, affirms that this manuscript is an honest, accurate, and transparent account of the study being reported; that no important aspects of the study have been omitted; and that any discrepancies from the study as planned have been explained.

## Data Availability

The data that support the findings of this study are available from the corresponding author upon reasonable request.
